# Effect of storage time on the silage quality and microbial community of mixed maize and faba bean in the Qinghai-Tibet Plateau

**DOI:** 10.3389/fmicb.2022.1090401

**Published:** 2023-01-19

**Authors:** Yafen Xin, Chen Chen, Yihao Zhong, Xingyue Bu, Shan Huang, Muhammad Tahir, Zhaochang Du, Weiguo Liu, Wenyu Yang, Jiayi Li, Yushan Wu, Zhengyong Zhang, Jinglong Lian, Qiyin Xiao, Yanhong Yan

**Affiliations:** ^1^College of Grassland Science and Technology, Sichuan Agricultural University, Chengdu, China; ^2^College of Agronomy, Sichuan Agricultural University, Chengdu, China; ^3^Agricultural Science Research Institute of Ganzi District, Garzê Tibetan Autonomous Prefecture, China

**Keywords:** mixed silage, microbial community, Qinghai-Tibet Plateau, maize, faba bean

## Abstract

Tibetan Plateau is facing serious shortage of forage in winter and spring season due to its special geographical location. Utilization of forages is useful to alleviate the forage shortage in winter and spring season. Consequently, the current study was aimed to evaluate the influence of storage time on the silage quality and microbial community of the maize (*Zea mays* L.) and faba bean (*Vicia faba* L.) mixed silage at Qinghai-Tibet Plateau. Maize and faba bean were ensiled with a fresh weight ratio of 7:3, followed by 30, 60, 90, and 120 days of ensiling. The results showed the pH value of mixed silage was below 4.2 at all fermentation days. The LA (lactic acid) content slightly fluctuated with the extension of fermentation time, with 33.76 g/kg DM at 90 days of ensiling. The AA (acetic acid) and NH_3_-N/TN (ammonium nitrogen/total nitrogen) contents increased with the extension of fermentation time and no significantly different between 90 and 120 days. The CP (crude protein) and WSC (water soluble carbohydrate) contents of mixed silage decreased significantly (*P* < 0.05) with ensiling time, but the WSC content remained stable at 90 days. The *Proteobacteria* was the predominant phyla in fresh maize and faba bean, and *Pseudomonas* and *Sphingomonas* were the predominant genera. After ensiling, *Lactobacillus* was the prevalent genus at all ensiling days. The relative abundance of *Lactococcus* increased rapidly at 90 days of ensiling until 120 days of fermentation. Overall, the storage time significant influenced the silage fermentation quality, nutrient content, and microbial environment, and it remained stable for 90 days of ensiling at Qinghai-Tibet Plateau. Therefore, the recommended storage time of forage is 90 days in Qinghai-Tibet Plateau and other cool areas.

## 1. Introduction

The Qinghai-Tibet Plateau is an important area of animal husbandry production in China, and yak is the foundation of generations of survival for farmers and herdsmen (Long, 2007; Wang et al., 2018). Due to the extreme climatic environment of high altitude, intense radiation, drought and cold on the Tibetan Plateau, the forage growth period is short and slow, and the total forage supply can hardly meet the annual feed demand of livestock (Yuan et al., 2013; Zhou et al., 2018). Maize (*Zea mays* L.) and faba bean (*Vicia faba* L.) can grow normally in the area, providing a high biological yield, and they can be preserved for yak winter and spring feeding. The traditional storage methods of baling, sun-curing and air-drying of forage grass are susceptible to climatic and environment, thereby, reducing the crude protein (CP) content, palatability and digestibility (Qin et al., 2013). Ensiling is a simple, easy and low cost storage method of forage (Chen et al., 2020), which can effectively preserve the nutritional value of forage (Wilkinson and Rinne, 2018; Li et al., 2022a), and alleviate the shortage of forage for livestock in spring and winter season in plateau areas.

Whole-plant maize silage has the characteristics of high yield and good palatability (Qu et al., 2013), and has become the main feed for dairy cows worldwide (Ferraretto et al., 2018). Moreover, particularly in comparison with other forages, whole-plant maize silage can provide high energy (mainly from starch in the kernel fraction) along with physically effective neutral detergent fiber (NDF, provided by the stover fraction) concurrently (Ferraretto et al., 2018; Li et al., 2022b). However, maize silage has low protein content and cannot meet protein requirements for livestock (Contreras-Govea et al., 2008). Faba bean is a cool-season, annual protein-rich cereal legume, which is an important source for both humans and livestock’s (Gu et al., 2020). A combination of legume with cereal silage has been proven successful because high protein content of legume enhances the nutritional value, while the abundant carbohydrates provided by cereal offer an enough substrate for lactic acid bacteria (LAB) to multiply, ensuring quality fermentation of mixed silages (Zhu et al., 2011; Zeng et al., 2020, 2022; Gülümser et al., 2021). However, little is known about maize and faba bean mixed silage in Qinghai-Tibet Plateau, which need further study to help livestock survive winter and spring seasons in this region.

Silage is a very complex process which involves microbial activity and biochemical changes (Xin et al., 2021). Generally, ensiling of forages is determined by many factors, such as forage species, storage environment, and moisture content. With the silage fermentation, the microbial community and forage nutrient content also will change. Generally, the appropriate temperature for silage fermentation ranged from 20 to 30°C (Zhou et al., 2016). Low temperatures can not only inhibit fermentation by reducing microbial growth rate and enzymatic activity, but also alter the composition of lactic acid bacteria (LAB) microbiota in different ecosystems by selecting specific species that can adapt to low growth temperatures (Zhou et al., 2016). In the actual production practice, under low temperature environment, the silage produces less acid (Kung, 2010), pH decreases slowly (Ali et al., 2015), and the number of bad microorganisms such as yeast is higher, which adversely affect the silage quality. The Qinghai-Tibet Plateau has higher altitude and low temperatures, which will have an important impact on fermentation process and the microbial community structure of silage. Moreover, under the special climate conditions of the Qinghai-Tibet Plateau, less information is available on the effect of fermentation time on the microbial and nutrient characteristics of mixed silage, and the optimal silage time is unclear.

Therefore, the current study was aimed to evaluate the response of chemical composition, fermentation quality, and microbial community of mixed maize and faba bean silage to different storage periods. The results could provide a theoretical support for future mixed silage production in the Qinghai-Tibet Plateau.

## 2. Materials and methods

### 2.1. Study site and sample collection

The experimental site was located in Daofu County, Kangding City, Sichuan Province, located on the southeastern edge of the Qinghai-Tibet Plateau (101°7′30″E, 30°58′46″N, altitude 3,450 m). This region has a plateau frigid-temperate climate, with sufficient sunshine, concentrated rainfall, large daily temperature difference and short frost-free period and other characteristics. The variety of silage maize (M) and faba bean (B) was Demeiya No.1 (growing period 110 days) and Italy Qimi (growing period 162 days), respectively. M and B were harvested with a 15 and 5 cm stubble height on 14 October 2020, respectively. At that time M was in milk stage and B was in pod stage. The materials were cut into 1–2 cm theoretical lengths. The M and B were evenly mixed with a 7:3 fresh weight (FM) ratio. The 300 g sample was immediately vacuum-sealed in polyethylene plastic bags (30 cm × 40 cm, China) for a total of 12 bags (4 storage times × 3 replicates) and kept at local outdoor environment. The 3 bags were opened on 30, 60, 90, and 120 days after ensiling, respectively. Chemical composition, fermentation quality, and bacterial community analysis were determined when bags were opened by treated fermentation days.

### 2.2. Chemical and fermentation compositions analysis

Fresh samples and ensiled materials were deenzymed at 105°C, then were dried at 65°C to constant weight to determine the dry matter (DM) content. The dried materials were stored after grinding and filtering with 1.0 mm sieve for subsequent analysis. The water-soluble carbohydrate (WSC) content was analyzed by anthrone-sulfuric acid colorimetric method (Owens et al., 1999). The CP and total nitrogen (TN) contens were detected using Dumas combustion method (Hwang et al., 2020). The NDF and acid detergent fiber (ADF) were determined according to the method of Van Soest et al. (1991). According to Yan et al. (2019), 20 g samples were evenly mixed in 180 mL reverse osmosis water, filtered with 4 layers of gauze, and the filtrate pH was determined using a glass electrode pH meter. The ammonia nitrogen (NH_3_-N) content was analyzed by the phenol-hypochlorite method (Li et al., 2021). Part of the filtrate from samples was centrifuged at 12,000 rpm for 10 min and passed 0.22 μm filter before lactic acid (LA), acetic acid (AA), propionic acid (PA) and butyric acid (BA) were analyzed by high phase liquid chromatography (HPLC, KC-811, Shimadzu Co., Kyoto, Japan). The setting parameters were as follows: The wavelength was set to 210 nm. The mobile phase was 3 mmol/L perchloric acid, with a column temperature of 50°C, and a flow rate of 0.5 mL/min.

### 2.3. Microbial population counting

Microbial population counts were the same as the previous report (Zeng et al., 2020). In brief, each 20 g sample was mixed to 180 mL sterile saline (0.85% NaCl). Serial dilutions were performed from 10^–1^ to 10^–7^. *Enterobacter* were cultivated on Violet Red Bile Agar (Difco, Hopebil, Qingdao, China) and counted after 24 h of aerobic growth at 37°C. LAB were cultivated on De Man, Rogosa, and Sharpe agar (Difco, Hopebil, Qingdao, China) and counted after 48 h of anaerobic growth at 37°C. Molds and Yeasts were determined by Potato Dextrose Agar (Difco, Hopebil, Qingdao, China) and counted after 72 h of aerobic growth at 28°C. Yeasts and molds were discriminated using colony appearance and cell morphology observations.

### 2.4. Bacterial community analysis

Microbial DNA was extracted by the M5 Plant Genomic DNA kit 200T (MF070-04, Mei5bio, Beijing, China) with the manufacturer’s protocol. The DNA purification was checked by a Nanorop microspectrophotometer (NanoDrop 2000, Thermo Fisher, USA), and DNA integrity was determined using 2% agarose gels electrophoresis, and the eligible samples were used for further analysis.

The DNA samples sequencing was performed at the Guangzhou Genedenovo Biotechnology Co., Ltd. After the genomic DNA was extracted, the 16S rDNA V5 + V7 region was amplified with specific primers with barcode. The primer sequence was: 799F (AACMGGATTAGATACCCKG) and 1193R (ACGTCATCCCCACCTTCC). The purified amplified product (amplicon) was connected to the sequencing connector to construct the sequencing library, and it was equimolar and paired-end sequenced (PE250) by Illumina platform. After raw data was obtained by sequencing, a large amount of low-quality data or no-biologically meaningful data is generated due to PCR and sequencing errors (such as chimeras). Therefore, we performed the following operations to ensure statistical reliability and biological effectiveness of the subsequent analysis. First, we filtered the low-quality reads by the FASTP software, and then performed the assembly. The two-end reads were spliced into tag by the FLASH software, and then the tag was filtered, and the resulting data was Clean tag. Next, the clustering was performed based on Clean tag through the UPARSE algorithm of the USEARCH software. Chimera tag was removed by the UCHIME algorithm of USEARCH software and the resulting data were Effective tag. After obtaining the OTU, the OTU abundance statistics were performed based on the Effective tag.

The indexes of Chao, Shannon, Simpson, and Goods coverage were analyzed in QIIME (version 1.9.1). The primary coordinates analysis (PCoA) was generated and plotted by the R project. The Venn analysis was performed by the R software VennDiagram package (version 1.6.16). Abundances statistics for every taxonomy were visualized through Krona (version 2.6). Microbial stacked bar plots were performed by the R project ggplot2 package (version 2.2.1). Species circular layout representations abundances were plotted through circos (version 0.69-3). The impact of environmental factors on community composition was performed by the R project Vegan software package (version 2.5.3). Heatmaps were generated using the dynamic real-time interactive online data analysis platform.^1^

### 2.5. Statistical analyses

Data collation and mapping were performed by using Excel 2019. Before statistical analysis, microbial population counting of samples were calculated in log_10_ cfu/g of FM. The data collected were subjected to the independent sample T test for fresh materials and one-way ANOVA for ensiling samples by SPSS 27.0 software. Significant differences existed if the probability level was below 0.05.

## 3. Results

### 3.1. Characteristics of fresh materials

Chemical composition and microbial population of the fresh maize and faba bean before ensiling are presented in Table 1. The DM content of M was 238.80 g/kg, higher than that of B (225.11 g/kg; *P* < 0.05). The WSC content of M reached 222.86 g/kg DM, and much higher than that of B (*P* < 0.05), while CP content was lower than that of B (*P* < 0.05), which is 83.97 g/kg DM. ADF and NDF contents of M were 241.90 and 491.98 g/kg DM, and those of B were 262.06 and 450.20 g/kg DM, respectively. Furthermore, LAB, yeasts, and *Enterobacter* population of M were higher than B (*P* < 0.05). No molds were detected in the M, and the population of molds was 5.15 log_10_ cfu/g FM in B.

**TABLE 1:** Chemical composition and microbial population before ensiling.

Treatment	M	B
**Chemical composition**		
Dry matter (g/kg)	238.80^a^	225.11^b^
Crude protein (g/kg DM)	83.97^b^	138.14^a^
Water soluble carbohydrate (g/kg DM)	222.86^a^	110.65^b^
Acid detergent fiber (g/kg DM)	241.90^b^	262.06^a^
Neutral detergent fiber (g/kg DM)	491.98^a^	450.20^b^
**Microbial population (log_10_ cfu/g of FM)**		
Lactic acid bacteria	5.74^a^	3.25^b^
Yeasts	5.93^a^	5.27^b^
Molds	ND	5.15
Enterobacter	6.75^a^	5.97^b^

DM, dry matter; FM, fresh material; cfu, colony-forming unit; ND, not detected; M, maize, B, faba bean. Different letters within the same line indicate statistically significant difference between the treatments (*P* < 0.05).

### 3.2. Fermentation quality and chemical composition of ensiling maize and faba bean

The fermentation quality and microbial population of different ensiling days are shown in Table 2. The pH values of silage for four fermentation days were below 4.2, but the significant lowest pH value was observed at 30 days (3.97, *P* < 0.05). The NH_3_-N/TN content increased during fermentation, while that of silages were no significant different on the 90 and 120 days (*P* < 0.05). The LA content slightly fluctuated with the extension of fermentation time and was highest at 30 days of ensiling (35.21 g/kg DM), and was lowest at 60 days of ensiling (26.86 g/kg DM). The AA content increased with the extension of fermentation time but there was no significant difference on 90 days (16.42 g/kg DM) and 120 days (17.04 g/kg DM) of ensiling. The PA content of 90 days was significantly lower than silage of other fermentation days (2.07 g/kg DM; *P* < 0.05). The BA content of silage for all fermentation days was relatively stable levels and less than 1% DM. The LAB population significantly decreased with prolonged fermentation time and was lowest on 120 days (6.85 log_10_ cfu/g of FM). Yeasts were only detected in silage for 60 days. Molds and *Enterobacter* were not detected in silage for all storage days.

**TABLE 2:** Fermentation characteristics and microbial population after ensiling.

	Ensiling days				SEM
	**30**	**60**	**90**	**120**	
**Fermentation characteristics**					
pH	3.97^c^	4.09^a^	4.00^b^	4.01^b^	0.01
NH_3_-N/TN	4.02^b^	4.88^b^	6.52^a^	6.55^a^	0.35
Lactic acid (g/kg DM)	35.21^a^	26.86^c^	33.76^a^	30.20^b^	1.12
Acetic acid (g/kg DM)	11.79^c^	12.97^b^	16.42^a^	17.04^a^	0.68
Propionic acid (g/kg DM)	2.95^a^	2.62^a^	2.07^b^	3.02^a^	0.13
Butyric acid (g/kg DM)	0.51	0.51	0.51	0.59	0.01
**Microbial population (log_10_ cfu/g of FM)**					
Lactic acid bacteria	8.65^a^	8.27^a^	7.42^b^	6.85^c^	0.22
Yeasts	ND	6.16	ND	ND	0.80
Molds	ND	ND	ND	ND	–
Enterobacter	ND	ND	ND	ND	–

NH_3_-N/TN, ammonium nitrogen/total nitrogen; DM, dry matter; FM, fresh material; cfu, colony-forming unit; ND, not detected; SEM, standard error of mean. Different letters within the same line indicate statistically significant difference between the treatments (*P* < 0.05).

DM of all silages varied from 221.13 to 230.37 g/kg (Table 3), and was lowest at 60 days (221.13 g/kg, *P* < 0.05). CP content decreased significantly with prolonged silage (*P* < 0.05). WSC content decreased markedly for all ensiling days with no differences in 90 and 120 days of fermentation (*P* < 0.05). ADF and NDF content in different ensiling times were significantly different (*P* < 0.05), and variation rang was 254.54 to 265.00 and 385.70 to 453.10 g/kg DM, respectively.

**TABLE 3:** Chemical composition after ensiling.

Treatment	Ensiling days				SEM
	**30**	**60**	**90**	**120**	
Dry matter (g/kg)	230.37^a^	221.13^c^	223.89^b^	225.21^b^	1.03
Crude protein (g/kg DM)	120.55^a^	109.11^b^	83.94^c^	52.96^d^	7.83
Water soluble carbohydrate (g/kg DM)	95.82^a^	74.80^b^	60.60^c^	59.40^c^	4.43
Acid detergent fiber (g/kg DM)	254.54^c^	260.50^b^	263.17^ab^	265.00^a^	1.31
Neutral detergent fiber (g/kg DM)	385.70^c^	462.70^a^	451.92^b^	453.10^b^	9.26

DM, dry matter; SEM, standard error of mean. Different letters within the same line indicate statistically significant difference between the treatments (*P* < 0.05).

### 3.3. Microbial community of fresh and ensiled samples

**FIGURE 1:**
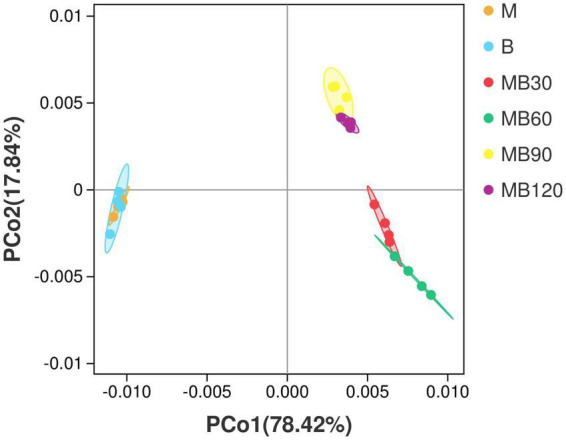
Principal coordinates analysis (PCoA) of bacterial communities for fresh materials and silage. M, maize; B, faba bean; MB30, 30 days of mixed silage of maize and faba bean; MB60, 60 days of mixed silage of maize and faba bean; MB90, 90 days of mixed silage of maize and faba bean; MB120, 120 days of mixed silage of maize and faba bean.

**FIGURE 2:**
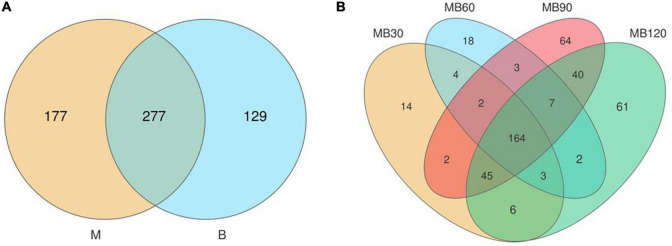
**(A)** Diagram of bacterial OTUs of fresh materials. **(B)** Venn diagram of bacterial OTUs of silage. M, maize; B, faba bean; MB30, 30 days of mixed silage of maize and faba bean; MB60, 60 days of mixed silage of maize and faba bean; MB90, 90 days of mixed silage of maize and faba bean; MB120, 120 days of mixed silage of maize and faba bean.

A total of 733,561 effective tags were generated using the high-throughput sequencing of the 16S rRNA gene for 8 fresh samples and 16 ensiled materials. The alpha-diversity of bacterial communities of the samples is listed in Table 4. The goods coverage were above 99% at all ensiling days. Silage decreased the Shannon, Simpson indexes, and Chao values relative to the fresh materials. Compared with the B, the higher Shannon, Simpson indexes, Chao values was observed in M. The result of principal coordinates analysis (PCoA; Figure 1) showed principal coordinates 1 (PCo1) and 2 (PCo2) explain 78.42 and 17.84% in the total variance for samples, respectively. There were clustered between M and B, MB30 and MB60, MB90 and MB120, and bacterial community was overlaps respectively. According to Venn analysis, the bacterial diversity of M was higher than that of B, and in addition, 277 overlapping bacterial OTUs in the M and B (Figure 2A). There were 164 overlapping bacterial OTUs in silage for all storage days (Figure 2B). A total 173 overlapping bacterial OTUs were detected at 30 and 60 days of ensiling. Furthermore, there were similar bacterial OTUs at 90 and 120 days of ensiling, and more bacterial OTUs were shared.

**TABLE 4:** Alpha-diversity of bacterial communities in fresh materials and after ensiling.

Treatment	Observed species	Shannon	Simpson	Chao	Goods coverage
M	379^a^	5.14^a^	0.95^a^	407.76^a^	0.9996
B	332^b^	5.10^a^	0.94^a^	351.64^b^	0.9997
MB30	262^c^	2.88^c^	0.68^d^	305.52^b^	0.9995
MB60	208^d^	2.82^c^	0.73^c^	244.8^c^	0.9996
MB90	310^b^	3.99^b^	0.89^b^	339.24^b^	0.9996
MB120	302^b^	3.87^b^	0.87^b^	331.4^b^	0.9997

M, maize; B, faba bean; MB30, 30 days of mixed silage of maize and faba bean; MB60, 60 days of mixed silage of maize and faba bean; MB90, 90 days of mixed silage of maize and faba bean; MB120, 120 days of mixed silage of maize and faba bean. Different letters within the same line indicate statistically significant difference between the treatments (*P* < 0.05).

**FIGURE 3:**
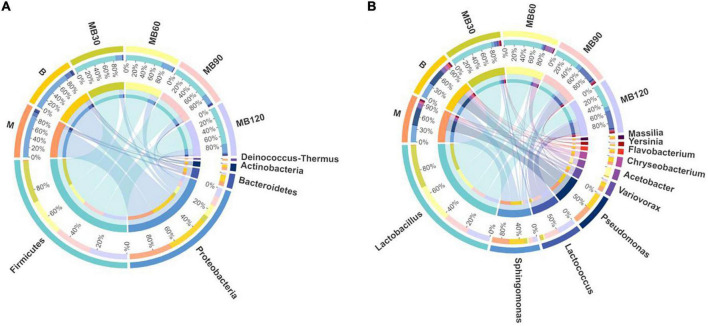
Circos map of bacterial communities at the phylum **(A)** and genus levels **(B)** for fresh materials and ensiling. M, maize; B, faba bean; MB30, 30 days of mixed silage of maize and faba bean; MB60, 60 days of mixed silage of maize and faba bean; MB90, 90 days of mixed silage of maize and faba bean; MB120, 120 days of mixed silage of maize and faba bean.

**FIGURE 4:**
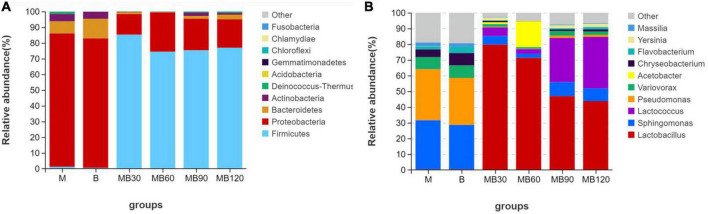
Relative abundance of bacterial communities at the phylum **(A)** and genus levels **(B)** for fresh materials and ensiling. M, maize; B, faba bean; MB30, 30 days of mixed silage of maize and faba bean; MB60, 60 days of mixed silage of maize and faba bean; MB90, 90 days of mixed silage of maize and faba bean; MB120, 120 days of mixed silage of maize and faba bean.

The bacterial communities of fresh materials and ensiling at the phylum and genus levels are shown in Figure 3 (Circos map) and Figure 4 (accumulation map). *Firmicutes and Proteobacteria* were the top two phylas of all treatments. *Lactobacillus*, *Sphingomonas*, *Pseudomonas*, and *Lactococcus* were the top four genera in all treatments. The dominant phyla of fresh materials in M and B were *Proteobacteria*, with relative abundances of 84.95 and 82.44%, respectively. The abundances of *Firmicutes* was the most abundant phylum after ensiling, exceeding 74% for all fermentation days while those of *Proteobacteria, Bacteroidetes* and *Actinobacteria* decreased significantly after ensiling (*P* < 0.05). At the genus level, a high abundances of both *Sphingomonas* and *Pseudomonas* were found in the M and B, with relative abundances exceeding 28%. In addition, *Variovorax* and *Chryseobacterium* were also existence in fresh materials. *Sphingomonas* and *Pseudomonas* relative abundances significantly reduced after ensiling, and *Lactobacillus* was the predominant genus after ensiling for all silages (*P* < 0.05). However, the relative abundance of *Lactobacillus* decreased with the extension of fermentation time, and was lowest at 120 days of ensiling (43.81%). The most abundant *Acetobacter* was observed at 60 days of ensiling (16.51%). *Sphingomona* existed in the whole silage period. The *Pseudomonas* abundances decreased to below 2% during ensiling. The *Lactococcus* abundances was lower within 90 days of ensiling, and then rapidly increased with increasing fermentation time until the 120 days of fermentation. Moreover, the *Sphingomona*, *Pseudomonas*, *Variovorax*, *Chryseobacterium*, *Flavobacterium*, *Yersinia*, and *Massilia* were the minor in the mixed silage after ensiling.

### 3.4. Association analysis of fermentation characteristics with bacterial community

**FIGURE 5:**
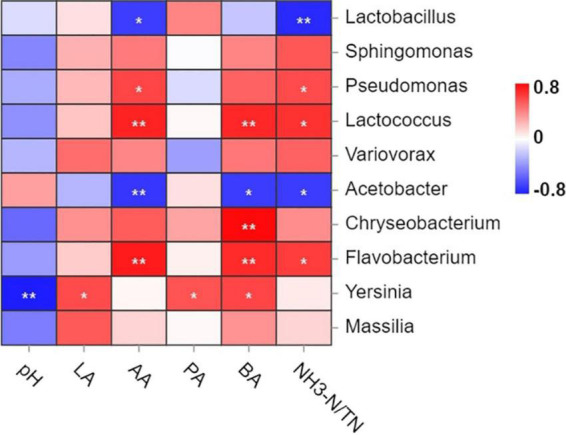
Spearman association analysis between bacterial abundance and fermentation products at the genus level. Red indicated a positive correlation, and blue indicated a negative correlation. LA, lactic acid; AA, acetic acid; PA, propionic acid; BA, butyric acid; NH_3_-N/TN, ammonium nitrogen/total nitrogen. *P < 0.05; **P < 0.01.

The spearman correlation analysis between fermentation products and bacterial abundance is listed in Figure 5. Specifically, *Yersinia* was negatively correlated (*P* < 0.01) with pH, while positively correlated with LA and PA contents (*P* < 0.05). The AA content was negatively correlated with *Acetobacter* (*P* < 0.01) and *Lactobacillus* (*P* < 0.05), and positively correlated with *Lactococcus, Flavobacterium* (*P* < 0.01) and *Pseudomonas* (*P* < 0.05). Negative correlation between *Acetobacter* and BA content was observed (*P* < 0.05). In contrast, most bacteria were related positively with BA content. Such as *Chryseobacterium* and *Flavobacterium* (*P* < 0.01). NH_3_-N content was negatively correlated with *Lactobacillus* (*P* < 0.01) and *Acetobacter* (*P* < 0.05).

## 4. Discussion

### 4.1. Effect of mixed silage on fermentation characteristics

The pH value is a key indicator to estimate the success of fermentation, and that is also always considered as a pivotal index to reflect microbial activity during ensiling (Kung et al., 2018; Li et al., 2022c). In the study, the pH value was below 4.2 at all stages of ensiling. Similarly, the same result was also observed in the mixed whole maize and soybean (Wen et al., 2022). This indicates that the northwest Sichuan plateau region provided a good fermentation environment for mixed silage of maize and legume. Moreover, low pH levels also restricted the growth of undesirable microorganisms such as *Enterobacteria*, which reduced the risk of azotate production. The NH_3_-N generally indicates the silage proteolysis during ensiling which is driven by plant enzymes protein degradation and microbial decomposition using proteins and amino acids, and is associated with *Clostridial* activity (Wang et al., 2019a). Briefly, fermentation resulted in an increase in NH_3_-N/TN of silage. A similar result was obtained by Naeini et al. (2014), who shown that sweet sorghum NH_3_-N content increased steadily for 30 days to 120 days of storage time. Newbold et al. (2006) reported increasing amount of NH_3_-N of the whole maize silage during 10 months. The mixed silage had a good and stable internal environment, which may be the reason for no significant in NH_3_-N/TN content after 90 days. The decrease in the LA content during ensiling might be due to decreased WSC content in the silage, resulting in slow fermentation of LAB (Zeng et al., 2020). Moreover, some anaerobic microorganisms could be present and decompose LA and produce AA or PA, leading to reduction in LA content (Shao et al., 2002). The high abundance of yeasts degraded LA to ethanol (Woolford, 1990), resulting lowest LA content in silage for 60 days and reduced nutritional. The AA can effectively inhibit yeast growth, improving the stability during ensiling and feeding processes, minimizing the silage nutrient loss during long-term storage (Li and Nishino, 2013). The AA content of mixed silage increased during fermentatiom indicating the activity of some hetero-fermentative LAB (Wang et al., 2021). The presence of PA is unpopular as they reflect the nutrient loss in the silage (Wang et al., 2021). Moreover, the growth of yeasts and molds can be inhibited effectively by PA (Oladosu et al., 2016). The BA content beyond 5 g/kg DM could affect the feed palatability and reduce feed intake in livestock (Muck, 2010). In this study, BA was detected in silage within reasonable range, indicting good fermentation quality of mixed maize and faba bean silage in cold regions.

### 4.2. Effect of mixed silage on microbial population

The LAB is known to produce large amounts of organic acids, lowering pH, inhibiting the growth of undesirable microbes, ultimately leading to quality silage (Ni et al., 2015). There were a large number of undesirable microorganisms such as yeast and *Enterobacter* in fresh maize and faba bean, which may lead to poor fermentation quality if both these crops ensiled alone. However, the epiphytic LAB count for fresh maize was higher 10^5^ cfu/g FM, which was sufficient to initiate LA fermentation in anaerobic conditions after mixing silage with faba bean. In the current study, the LAB population significantly decreased with prolonged fermentation time and was lowest on 120 days, which might be related to the insufficient substrates such as WSC (Wang et al., 2021). Moreover, it was quite fascination to found that a large number of yeasts existed in mixed silage after 60 days of ensiling. Yeasts is an aerobic microorganisms which competed with LAB for WSC, causing rise in pH and substantial DM loss of silage (Spoelstra et al., 1988; Lv et al., 2020). However, the molds and *Enterobacter* were not detected after ensiling in this study which might be related to the enough acid or lower pH of silage to inhibit the growth of these microbes.

### 4.3. Effect of mixed silage on chemical composition

The DM content is considered as crucial index of nutritional preservation of forages (Hu et al., 2009). The DM content of fresh maize (238.80 g/kg DM) was relatively low, and DM content of faba bean (225.11 g/kg DM) was similar reported of Li et al. (2022a), while was greater than report of Palmio et al. (2022). This might be attributed to the agronomic practices, difference in varieties, and growing environment of forages. Moreover, due to the lower temperature and early frost season on the Qinghai-Tibet Plateau, the DM content unable to increase by delayed harvest. The DM content of mixed silage at all ensiling days was ranged from 221 to 231 g/kg after ensiling, which was lower than the ideal level of 300–350 g/kg (Guyader et al., 2018). High moisture content can easily lead to large growth of *Clostridium*, resulting unsuccessful silage (Li et al., 2014). In this study, rapid acidification caused by the production of organic acid and along with lower temperature of plateau region limited growth of *Clostridium*. In addition, the higher nutritive value is expected when forage crops are grown under the cool conditions encountered at higher latitudes as reported by Buxton (1996). In this study, the WSC content of fresh maize and faba bean beyond the 6% which was sufficient to initiate the silage fermentation of mixed silage (Guo et al., 2021). The high CP content of faba bean can achieve a better fermentation quality of mixed silage. Microbial depletion and degradation lead to reduced WSC after ensiling (Sariçiçek et al., 2016). At 60 days of silage, mixed silage WSC decreased was due to the fact undesired bacterial proliferation increased nutrient loss. The higher NDF content shortened the degradation time of feed in the rumen and accelerated the flow rate of feed in the gastrointestinal tract, thus reducing the whole intestinal digestibility of DM, CP, etc. (Shi et al., 2015). The CP content of silage decreased from 120.55 g/kg for 30 days to 52.96 g/kg for 120 days. Sariçiçek et al. (2016) reported that CP content of maize silage decreased from 89.5 g/kg for 90 days to 65.40 g/kg for 202 days during fermentation, which was in accordance with our consequence. This decrease might be associated with the degradation of some nutrients by microorganisms in anaerobic conditions (Sariçiçek et al., 2016). But our results showed more protein degradation. The differences in studies might be related to the addition of faba bean and silage conditions.

### 4.4. Effect of mixed silage on microbial community

The alpha-diversity is adopted to estimate the richness, diversity, and evenness of species in bacterial communities (Chi et al., 2022). The goods coverage was above 99% in all treatments, indicating the sequencing adequately represented the real situation for the bacterial community. Compared to the epiphytic microorganisms on fresh faba bean, fresh maize existed more bacterial diversity and richness. After ensiling, the mixed silages had lower Shannon and Simpson indexes relative to the fresh materials which indicated that bacterial diversity decreased after ensiling. Similarly, Polley et al. (2007) reported that when the advantageous bacteria were abundant, the diversity of microbial communities was reduced. The bacterial diversity was also reduced after ensiling of wheat and Italian ryegrass (Keshri et al., 2019; Yan et al., 2019). The beneficial microorganisms such as *Lactobacillus* begin to grow well under anaerobic conditions, which reduce the pH by producing sufficient LA and inhibit the growth of undesirable microorganisms leading to a significant reduction in microbial diversity of silage during fermentation. The PCoA and venn analysis indicated that the bacterial population was overlapped between M and B, MB30 and MB60, MB90 and MB120, respectively. This highlights that ensiling time had significant effects on the bacterial community succession. A stable fermentation environment enhanced antimicrobial activity and then changed the bacterial activity of silage with prolonged ensiling time (Ravi Kanth Reddy et al., 2020). Therefore, succession in microbial communities would significantly affect the silage quality during fermentation.

Natural fermentation is dependent on epiphytic microbial communities, which vital role during the whole fermentation process (Xu et al., 2018). Therefore, understanding the composition of plant epiphytic microbial communities is of great importance, and could be better explore the silage fermentation process. In the present study, *Proteobacteria* was the dominant phyla in fresh materials, with relative abundances higher than 82%. *Sphingomonas* and *Pseudomonas* dominated the microbial composition in fresh maize and faba bean before ensiling. Many harmful microorganisms attached to the fresh materials could easy to compete with LAB and caused nutrient loss in the silage. Similary, Xu et al. (2018) reported that *Agrobacterium*, *Microbacterium*, *Sphingobacterium*, and *Chryseobacterium* were the dominant bacteria before ensiling of the whole crop corn. The dominant bacteria in fresh faba bean were *Sphingomonas*, *Lactobacillus*, unidentified*_Chloroplats*, and *Pseudomonas* (Li et al., 2022a). The differences in these studies indicted that the bacterial colonization of the plant surfaces depended on many factors, including plant species, climatic characteristics, solar radiation intensity, etc (Cai et al., 1999; McGarvey et al., 2013). Although the microorganisms attached in fresh materials would be affected by the external environment, but the undesirable microorganisms were still more abundant. There were similar microbial community structure and 277 overlapping bacterial OTUs in the maize and faba bean, confirming that the environment significantly affected plant epiphytic microorganisms. After ensiling, most undesired microorganisms were inhibited under anaerobic conditions. This was well confirmed in this study as *Lactobacillus* was predominant genus in mixed silage after ensiling. In addition, antibacterial substances such as LA and AA inhibited harmful microorganisms during ensiling, and the relative abundances of *Sphingomonas* and *Pseudomonas* genera were decreased.

An insight into the bacterial species and relative abundances during fermentation is helpful to evaluate the fermentation quality and to improve the preservation of silage (Guan et al., 2018). This study showed that the abundance of genus *Firmicutes* increased significantly after ensiling. At the genus level, *Lactobacillus* became the dominant genus in mixed silage after ensiling. This might be because of the anaerobic conditions that facilitated the growth of LAB strains, which could produce LA to enhance their competitiveness by inhibiting other background bacteria (Wang et al., 2019b; Zhang et al., 2019). However, it was quite fascinating to found that, the *Lactobacillus* relative abundance decreased significantly after 60 days of ensiling, while the relative abundance of *Lactococcus* increased significantly in later phase of fermentation. This might be the *Acetobacter* remarkably effected silage fermentation by reconstructing the microbial community. *Acetobacter* is an obligate aerobic bacterium with the ability to partially oxidize alcohol or sugar into organic acids such as AA or gluconicacid (Xian et al., 2022). Therefore, it may be useful to inhibit the propagation of fungi and to prolong the aerobic stability of silage. Gao et al. (2018) detected *A. lovaniensis* in whey-based kefir beverages after Bod ljong cheese-making from the Qinghai-Tibet Plateau, and the study demonstrated the important role of *Acetobacter* in the flavor transformation process of whey beverages. In this study, *A. lovaniensis* was also detected in silage (see Supplementary Figure 1), perhaps the cold environment on the Tibetan Plateau is the reason for proliferation of *Acetobacter* in the later phase of fermentation. However, the prevalence of *Acetobacter* seemed to be very controversial in the silage. Because the *Acetobacter* acted simultaneously with yeasts and might cause the poor quality of maize silage, but the cause had not bean fully understood (Spoelstra et al., 1988). In view of the high relative abundance of *Acetobacter* in later phase of silage of this area, attention should be paid to further research. *Lactococcus* belongs to facultative anaerobes, which tended to dominate in early storage of silage, and their growth and reproduction were inhibited in anaerobic environment of silage (Wang et al., 2019c; Zeng et al., 2020). However, the *Lactococcus* abundances was rapidly increased and remained stable at 120 days of ensiling. It may be that the proliferation of *Acetobacter* promoted the growth of *Lactococcus* at 90 and 120 days, thus reducing the abundance of genus *Lactobacillus*, which was consistent with the changes of pH and LA, suggesting that *Lactococcus* is more competitive than *Lactobacillus* after changed silage environment. Meanwhile, it also caused the proliferation of *Sphingomonas*. As members of *Proteobacteria*, *Pseudomonas*, *Variovorax*, *Chryseobacteriumm*, *Flavobacterium, and Massilia* had lower relative abundances during ensiling. This is likely that the lower pH levels throughout the fermentation and anaerobic environment inhibited their growth (Bolsen et al., 1996), thus reducing their adverse effect on the silage quality.

### 4.5. Correlation analysis of bacterial community and fermentation products

To explore the correlations between fermentation products and microorganisms, the spearman association analysis was performed. The results indicated a significant correlation existed between microbial community structure and silage fermentation. As an important and key dominant bacteria in silage fermentation, *Lactobacillus* could effectively inhibit NH_3_-N production, thus preserving the nutrients of silage, consistent with previous results (Li et al., 2022a). *Lactobacillus* was negatively correlated with AA content, mainly due to the decreased relative abundance of genus *Lactobacillus* in late silage storage. The AA content increased with prolonged silage fermentation, and the rapid proliferation of *Lactococcus* at 60 and 120 days of ensiling contributed to the reduction of pH levels, which significantly inhibited the growth of spoilage bacteria such as yeast. Furthermore, positive correlations between NH_3_-N and the *Pseudomonas* was found. *Pseudomona* is inhibited at pH 4.5, using ammonium ions or nitrate as nitrogen source (Palleroni, 2015). Zeng et al. (2020) detected high abundance of genus *Acetobacter* upon the aerobic exposure phase. This study was mainly detected at 60 days of silage. *Acetobacter* activity consumed a certain amount of WSC (Xian et al., 2022), which led to the increase of the silage pH, and reduced the silage nutritional value. *Chryseobacterium*, *Flavobacterium*, and *Yersinia* were associated with aerobic spoilage in silage. *Chryseobacterium* had a certain ability to decompose protein and fat, and had the ability to cause rot on raw milk (Mwanza et al., 2022). *Flavobacterium* was widely found in water environments and was the main pathogenic bacteria of a variety of species of fish, with few reports in silage. *Yersinia* belongs to the family *Enterobacteriaceae*, which can ferment glucose and other carbohydrates to produce acids, most of which can reduce nitrate to nitrite (Shao et al., 2009), and grow and reproduce rapidly at low temperatures. However, the relative abundance of these bacteria was relatively lower in mixed silage. The correlation analysis suggested that there might be synergistic or antagonistic effects between metabolites and the growth and reproduction of different microorganisms, which was the similar to Xu et al. (2018).

## 5. Conclusion

The study confirmed that the benefits of mixing maize with faba bean on Tibetan Plateau. The prolonged storage time caused changes of the chemical composition fermentation characteristics of the silage. The mixed silage on 90 days of ensiling had the higher LA and AA contents, and the pH values, NH_3_-N, WSC, NDF, and ADF contents remained stable compared to 120 days of ensiling. Although the bacterial community varied with silage fermentation, *Lactobacillus* was the dominant bacterium and remained stable after 90 days of ensiling. Overall, mixed maize and faba bean silage had better fermentation quality and stable silage environment after 90 days of ensiling. Therefore, the recommended storage time of forage is 90 days in Qinghai-Tibet Plateau and other cool areas.

## Data availability statement

The datasets presented in this study can be found in online repositories. The names of the repository/repositories and accession number(s) can be found below: https://www.ncbi.nlm.nih.gov/, PRJNA895973.

## Author contributions

WL, WY, QX, and YY designed the study. YZ, XB, SH, ZD, ZZ, and YX performed the experiment. YZ, JYL, and YX analyzed the data. YX wrote the original manuscript. CC, MT, JLL, YW, and YY improved the manuscript. All authors reviewed and agreed to the published version of the manuscript.

## Footnotes

1
http://www.omicsmart.com

## Supplementary material

Supplementary Figure 1 Relative abundance of bacterial communities at the species levels for fresh materials and ensiling. M, maize; B, faba bean; MB30, 30 days of mixed silage of maize and faba bean; MB60, 60 days of mixed silage of maize and faba bean; MB90, 90 days of mixed silage of maize and faba bean; and MB120, 120 days of mixed silage of maize and faba bean.
