# Corrigendum: Effect of storage time on the silage quality and microbial community of mixed maize and faba bean in the Qinghai-Tibet Plateau

**DOI:** 10.3389/fmicb.2023.1161337

**Published:** 2023-03-29

**Authors:** Yafen Xin, Chen Chen, Yihao Zhong, Xingyue Bu, Shan Huang, Muhammad Tahir, Zhaochang Du, Weiguo Liu, Wenyu Yang, Jiayi Li, Yushan Wu, Zhengyong Zhang, Jinglong Lian, Qiyin Xiao, Yanhong Yan

**Affiliations:** ^1^College of Grassland Science and Technology, Sichuan Agricultural University, Chengdu, China; ^2^College of Agronomy, Sichuan Agricultural University, Chengdu, China; ^3^Agricultural Science Research Institute of Ganzi District, Garzê Tibetan Autonomous Prefecture, China

**Keywords:** mixed silage, microbial community, Qinghai-Tibet Plateau, maize, faba bean

In the published article, there was an error in the **Funding** statement. The grant number for the Sichuan Science and Technology Department Programs was displayed as “2021YFN0021”. The correct **Funding** statement appears below.

