# Supplementary figures and images for: Effect of storage time on the silage quality and microbial community of mixed maize and faba bean in the Qinghai-Tibet Plateau
Source: Front Microbiol. 2023 Jan 19;13:1090401. doi: 10.3389/fmicb.2022.1090401 (PMC9893498; doi:10.3389/fmicb.2022.1090401)

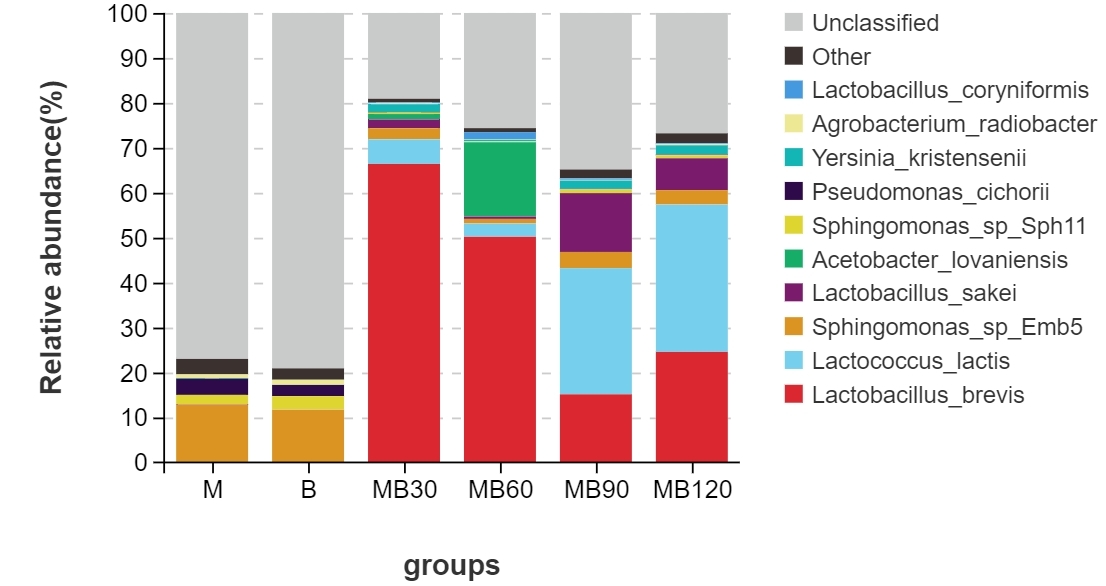

Supplement: Supplementary Figure 1 — Relative abundance of bacterial communities at the species levels for fresh materials and ensiling. M, maize; B, faba bean; MB30, 30 days of mixed silage of maize and faba bean; MB60, 60 days of mixed silage of maize and faba bean; MB90, 90 days of mixed silage of maize and faba bean; and MB120, 120 days of mixed silage of maize and faba bean. [file Image_1.jpg]
